# The Physician Himself, and Things That Concern His Reputation and Success

**Published:** 1890-02

**Authors:** 


The Physician Himself, and Things that Concern his Reputation and Success. By D. W. Cathell, M. D., Baltimore, Md. The ninth Edition, Revised and Enlarged. Philadelphia and London. F. A. Davis, Publisher. 1889.

This interesting work of 290 large octavo pages comes to us improved and enlarged from former editions. It has been well received by the profession. It is not only interesting, but instructive, and replete with useful and important suggestions for the practitioner, embodying an able and practical reply to such questions as these: "How shall I conduct myself as a physician, and what honorable means can I employ, in addition to scientific knowledge and book learning, in order to make my success more certain, more rapid and more complete, and to excel in the great battle of life to the fullest extent that lies in me?"

The book is in ten chapters under the following interesting heads:

1. "Things small in themselves have often a far-reaching significance."

2. "Who does the best his circumstances allow, does well, acts nobly; angels could do no more."

3. "Whatever a man soweth, that shall he also reap."

4. "The first step to wisdom is to be exempt from folly."

5. "A mind fraught with integrity is the most august possession."

6. "He is most free from danger who, even when safe, is on his guard."

7. "The successful man is the man who knows human nature as well as his profession."

8. "The wise and brave conquer difficulties by daring to attempt them."

9. "Together let us beat this ample field,
Try what the open, what the covert yield."

10. "Behold how good and how pleasant it is for brethren to dwell together in unity." R. C. W.

